# Supplementary material for: The Impact of Purifying and Background Selection on the Inference of Population History: Problems and Prospects
Source: Mol Biol Evol. 2021 Feb 16;38(7):2986–3003. doi: 10.1093/molbev/msab050 (PMC8233493; doi:10.1093/molbev/msab050)
Supplement: msab050_Supplementary_Data [file msab050_supplementary_data.pdf]

Supplementary Information

Supp Table 1: Performance of *fastsimcoal2* under neutrality and demographic equilibrium with varying chromosome sizes, when the correct model was specified. Inference was performed using 50 diploid individuals and all SNPs, and a comparison is given between simulations performed in SLiM (3.1) as well as msprime (0.7.3). The number of replicates for each chromosome size was set to 100, except for 1Gb chromosomes simulated in SLiM for which we report 10 replicates. Detailed methods including command lines can be found here: https://github.com/paruljohri/demographic_inference_with_selection/blob/main/CommandLines/SuppTable1.txt.

Simulated in SLiM 3.1			
**Chromosome size**	**True population size**	**Inferred population size**	
		**Mean**	**SD**
1 Mb	5000	5010	320
10 Mb	5000	5005	118
50 Mb	5000	4995	65
200 Mb	5000	4992	30
1 Gb	5000	4988	18
Simulated in msprime 0.7.3			
1 Mb	5000	5043	345
10 Mb	5000	5004	109
50 Mb	5000	5015	57
200 Mb	5000	5003	29
1 Gb	5000	5000	17

Supp Table 2: Model selection in *fastsimcoal2* for 100 replicates, in which the true model was neutral equilibrium and selection was performed with 4 models: equilibrium, instantaneous size change, exponential size change, and instantaneous bottleneck. Varying densities of SNPs (all SNPs, 1 per 5 kb, 1 per 50 kb and 1 per 100 kb) were used to perform inference. Simulations were performed using SLiM (3.1) for 10*N* generations. Detailed methods including command lines can be found here: https://github.com/paruljohri/demographic_inference_with_selection/blob/main/CommandLines/SuppFigure3_SuppTable2_5.txt.

Density of SNPs	Genome length	Equilibrium	Exponential change	Instantaneous change	Instantaneous Bottleneck	Total number of replicates
all	1Mb	33	11	18	38	100
	10Mb	32	17	27	24	100
	50Mb	16	25	29	30	100
	200Mb	5	10	41	44	100
	1Gb	0	4	3	3	10
1 per 5 kb	1Mb	69	7	9	15	100
	10Mb	49	8	18	25	100
	50Mb	10	4	35	51	100
	200Mb	0	0	48	52	100
	1Gb	0	0	5	5	10
1 per 50 kb	1Mb	83	7	4	6	100
	10Mb	83	5	4	8	100
	50Mb	59	10	10	21	100
	200Mb	20	6	40	34	100
	1Gb	0	0	4	6	10
1 per 100 kb	1Mb	86	4	3	7	100
	10Mb	78	5	7	10	100
	50Mb	64	6	14	16	100
	200Mb	43	6	30	21	100
	1Gb	0	1	5	4	10

Supp Table 3: Model selection in *fastsimcoal2* for 100 replicates, in which the true model was neutral equilibrium and selection was performed with 4 models: equilibrium, instantaneous size change, exponential size change, and instantaneous bottleneck. Varying densities of SNPs (all SNPs, 1 per 5 kb, 1 per 50 kb and 1 per 100 kb) were used to perform inference. Simulations were performed using msprime (0.7.3). Detailed methods including command lines can be found here:
https://github.com/paruljohri/demographic_inference_with_selection/blob/main/CommandLines/SuppFigure4_SuppTable3_6.txt.

Density of SNPs	Genome length	Equilibrium	Exponential change	Instantaneous change	Instantaneous Bottleneck	Total number of replicates
all	1Mb	29	16	18	37	100
	10Mb	22	12	31	35	100
	50Mb	13	18	38	31	100
	200Mb	11	8	31	40	100
	1Gb	5	12	26	57	100
1 per 5 kb	1Mb	63	3	12	22	100
	10Mb	42	10	19	29	100
	50Mb	4	7	42	47	100
	200Mb	1	2	55	42	100
	1Gb	0	1	66	33	100
1 per 50 kb	1Mb	83	4	6	7	100
	10Mb	80	4	6	10	100
	50Mb	61	9	12	18	100
	200Mb	19	6	29	46	100
	1Gb	0	2	48	50	100
1 per 100 kb	1Mb	89	3	3	5	100
	10Mb	86	4	4	6	100
	50Mb	70	3	11	16	100
	200Mb	47	5	20	28	100
	1Gb	0	3	47	50	100

Supp Table 4: Linkage disequilibrium (r^2) summarized in varying chromosomal sizes, and sampled in different densities, calculated using *Pylibseq* 0.2.3 in non-overlapping sliding windows across chromosomes with ~10 SNPs per window. SNPs from separate chromosomes represent completely unlinked SNPs – 1 SNP was randomly sampled from each of 100 separate replicate chromosomes simulated, and this random sampling was performed 100 times. Detailed methods including command lines can be found here: https://github.com/paruljohri/demographic_inference_with_selection/blob/main/CommandLines/SuppTable4.txt.

		**Number of SNPs per window**		r^2	
**Chromosome size**	**SNP density**	**mean**	**SD**	**mean**	**SD**
**10Mb**	all	10.7	3.6	0.1181	0.0885
	1 per 5 kb	8.3	0.7	0.0731	0.0504
	1 per 50 kb	9.8	0.4	0.0271	0.0141
	1 per 100 kb	10.0	0.2	0.0203	0.0101
	separate chromosomes	10.5	0.5	0.0103	0.0047
**1 Mb**	all	10.7	3.6	0.1174	0.0889
	1 per 5 kb	8.4	0.7	0.0725	0.0503
	1 per 50 kb	10.0	0.0	0.0252	0.0108
	1 per 100 kb	10.0	0.0	0.0193	0.0080
	separate chromosomes	10.5	0.5	0.0102	0.0047
**50Mb**	all	10.7	3.6	0.1181	0.0888
	1 per 5 kb	8.3	0.7	0.0729	0.0502
	1 per 50 kb	9.8	0.4	0.0269	0.0136
	1 per 100 kb	9.9	0.3	0.0203	0.0099
	separate chromosomes	10.5	0.5	0.0101	0.0046
**200 Mb**	all	10.6	3.6	0.1180	0.0888
	1 per 5 kb	8.3	0.7	0.0728	0.0499
	1 per 50 kb	9.8	0.4	0.0269	0.0137
	1 per 100 kb	9.9	0.3	0.0202	0.0098
	separate chromosomes	10.5	0.5	0.0101	0.0046
**1 Gb**	all	10.6	3.6	0.1181	0.0891
	1 per 5 kb	8.4	0.7	0.0727	0.0495
	1 per 50 kb	9.8	0.4	0.0269	0.0134
	1 per 100 kb	9.9	0.3	0.0204	0.0102
	separate chromosomes	6.5	0.5	0.0092	0.0046

Supp Table 5: Model selection with a higher AIC penalty (=25×the number of parameters) in which the true model was neutral equilibrium, and model selection was performed with 4 models: equilibrium, instantaneous size change, exponential size change, and instantaneous bottleneck. Varying densities of SNPs (all SNPs, 1 per 5 kb, 1 per 50 kb and 1 per 100 kb) were used to perform inference. Simulations were performed in SLiM (3.1) for 10*N* generations**.** Detailed methods including command lines can be found here: https://github.com/paruljohri/demographic_inference_with_selection/blob/main/CommandLines/SuppFigure3_SuppTable2_5.txt.

Density of SNPs	Genome length	Equilibrium	Exponential change	Instantaneous change	Instantaneous Bottleneck	Total number of replicates
all	1Mb	98	2	0	0	100
	10Mb	100	0	0	0	100
	50Mb	98	0	2	0	100
	200Mb	99	0	0	1	100
	1Gb	5	2	2	1	10
1 per 5 kb	1Mb	100	0	0	0	100
	10Mb	100	0	0	0	100
	50Mb	99	0	1	0	100
	200Mb	46	0	27	27	100
	1Gb	0	0	5	5	10
1 per 50 kb	1Mb	100	0	0	0	100
	10Mb	100	0	0	0	100
	50Mb	100	0	0	0	100
	200Mb	100	0	0	0	100
	1Gb	9	0	0	1	10
1 per 100 kb	1Mb	100	0	0	0	100
	10Mb	100	0	0	0	100
	50Mb	100	0	0	0	100
	200Mb	100	0	0	0	100
	1Gb	10	0	0	0	10

Supp Table 6: Model selection with a higher AIC penalty (=25×the number of parameters) in which the true model was neutral equilibrium, and model selection was performed with 4 models: equilibrium, instantaneous size change, exponential size change, and instantaneous bottleneck. Varying densities of SNPs (all SNPs, 1 per 5 kb, 1 per 50 kb and 1 per 100 kb) were used to perform inference. Simulations were performed using msprime (0.7.3). Detailed methods including command lines can be found here: https://github.com/paruljohri/demographic_inference_with_selection/blob/main/CommandLines/SuppFigure4_SuppTable3_6.txt.

Density of SNPs	Genome length	Equilibrium	Exponential change	Instantaneous change	Instantaneous Bottleneck	Total number of replicates
all	1Mb	100	0	0	0	100
	10Mb	100	0	0	0	100
	50Mb	97	0	2	1	100
	200Mb	96	0	4	0	100
	1Gb	93	1	3	3	100
1 per 5 kb	1Mb	100	0	0	0	100
	10Mb	100	0	0	0	100
	50Mb	97	1	0	2	100
	200Mb	45	1	30	24	100
	1Gb	0	1	66	33	100
1 per 50 kb	1Mb	100	0	0	0	100
	10Mb	100	0	0	0	100
	50Mb	100	0	0	0	100
	200Mb	100	0	0	0	100
	1Gb	89	1	4	6	100
1 per 100 kb	1Mb	100	0	0	0	100
	10Mb	100	0	0	0	100
	50Mb	100	0	0	0	100
	200Mb	100	0	0	0	100
	1Gb	100	0	0	0	100

Supp Table 7: Parameters underlying the human-like demographic models considered.

	Demographic models	Ancestral population size (N_{anc})	Current population size (N_{cur})	Time of change in generations
1	Equilibrium	10,000	10,000	NA
2	Exponential growth	1000	30,000	850
3	Instantaneous decline	12,300	2,100	4,750

Supp Table 8: Comparison of inference of parameters using *fastsimcoal2* under neutrality when inference is performed using all SNPs *vs*. using sparser SNP sampling (1 per 5 kb). In this example, 20% of the genome is exonic, and these exonic regions were masked when performing inference.

Demographic model	Parameter	True value	Inferred value using all SNPs (Mean±SD)	Inferred value using 1 SNP per 5 kb (Mean±SD)
Equilibrium	N_{anc}	10,000	9,781 ± 23	9,378 ± 567
Exponential growth	N_{anc}	1000	977 ± 3	963 ± 4
	N_{cur}	30,000	25,489 ± 123	25,641 ± 184
	Time of change	850	808 ± 3	816 ± 4
Instantaneous decline	N_{anc}	12,300	11,978 ± 463	11,513 ± 894
	N_{cur}	2,100	2,097 ± 15	2,681 ± 12
	Time of change	4,750	4,703 ± 246	7,458 ± 596

Supp Table 9: Nucleotide diversity in the presence of BGS relative to that under neutrality (B), calculated for a neutral site distance y bases from the end of a gene/exon of length 500 bp. The exon experiences purifying selection with strength $2N_e s$=10, where N_e is the effective population size and s is the reduction in fitness. Shown below is $t=hs$ where h is the dominance coefficient (assumed to be 0.5 here), r is the recombination rate per site per generation, and u is the mutation rate per site per generation. B was calculated using Equation 2 of Johri *et al*. (2020).

N_e	t ($2N_e s$ =10)	r	u	B (y=1)	B (y=10)	B (y=1000)
10^4	0.00025	1.00×10^{-8}	1.00×10^{-8}	0.9805	0.9806	0.9820
		1.00×10^{-6}	1.00×10^{-6}	0.5152	0.5312	0.9444
10^6	2.5×10^{-6}	1.00×10^{-8}	1.00×10^{-8}	0.5152	0.5312	0.9445
		1.00×10^{-6}	1.00×10^{-6}	0.4920	0.8227	0.9992

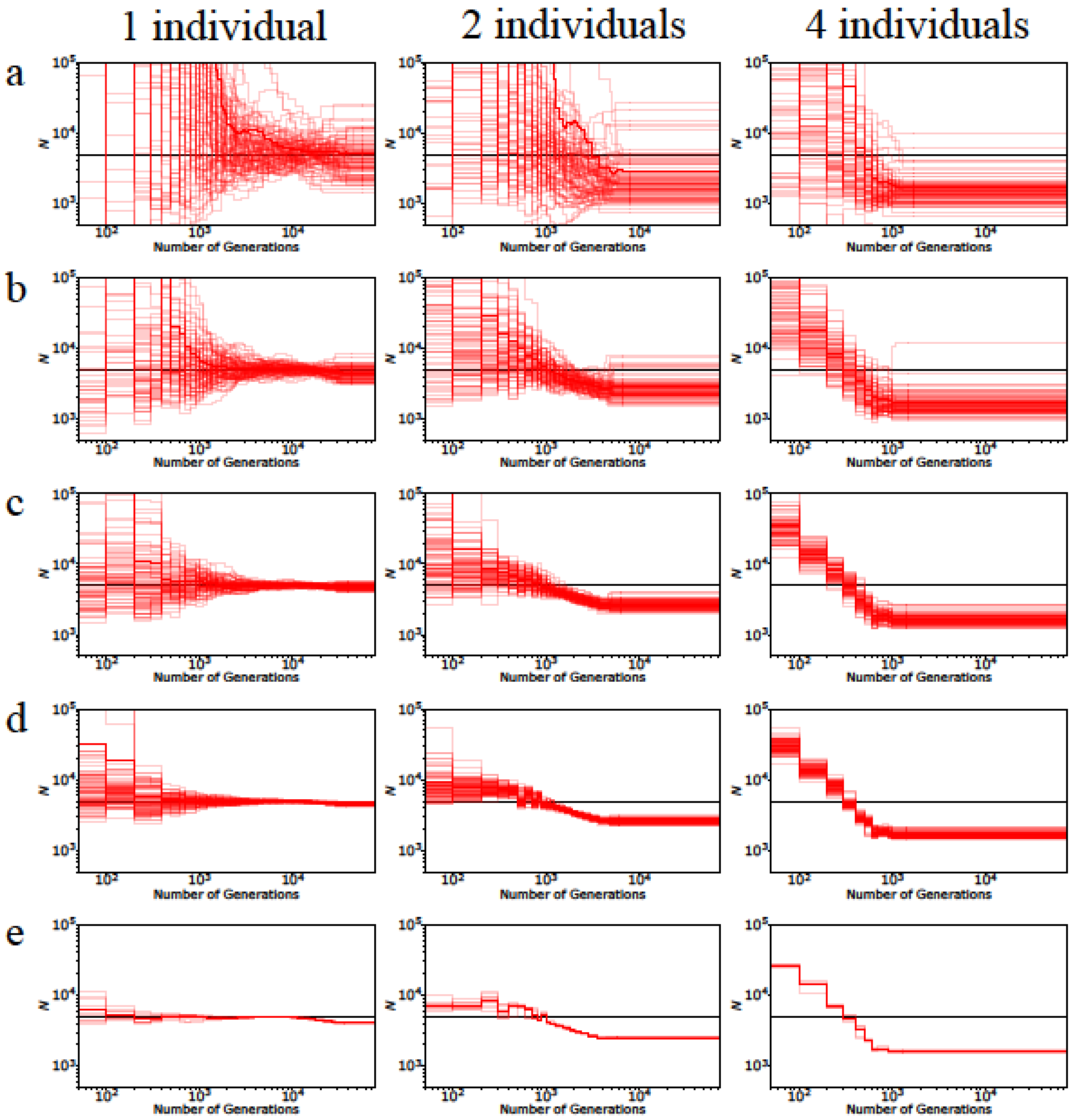

Supp Figure 1: Performance of MSMC under neutrality and demographic equilibrium when using 1, 2, and 4 diploid individuals for inference, for varying chromosome sizes: (a) 1Mb, (b) 10 Mb, (c) 50 Mb, (d) 200 Mb, (e) 1 Gb. Simulations were performed using SLiM (3.1) for 10*N* generations with 100 replicates for panels (a-d) and 10 for panel (e). Detailed methods including command lines can be found here: https://github.com/paruljohri/demographic_inference_with_selection/blob/main/CommandLines/SuppFigure1.txt.

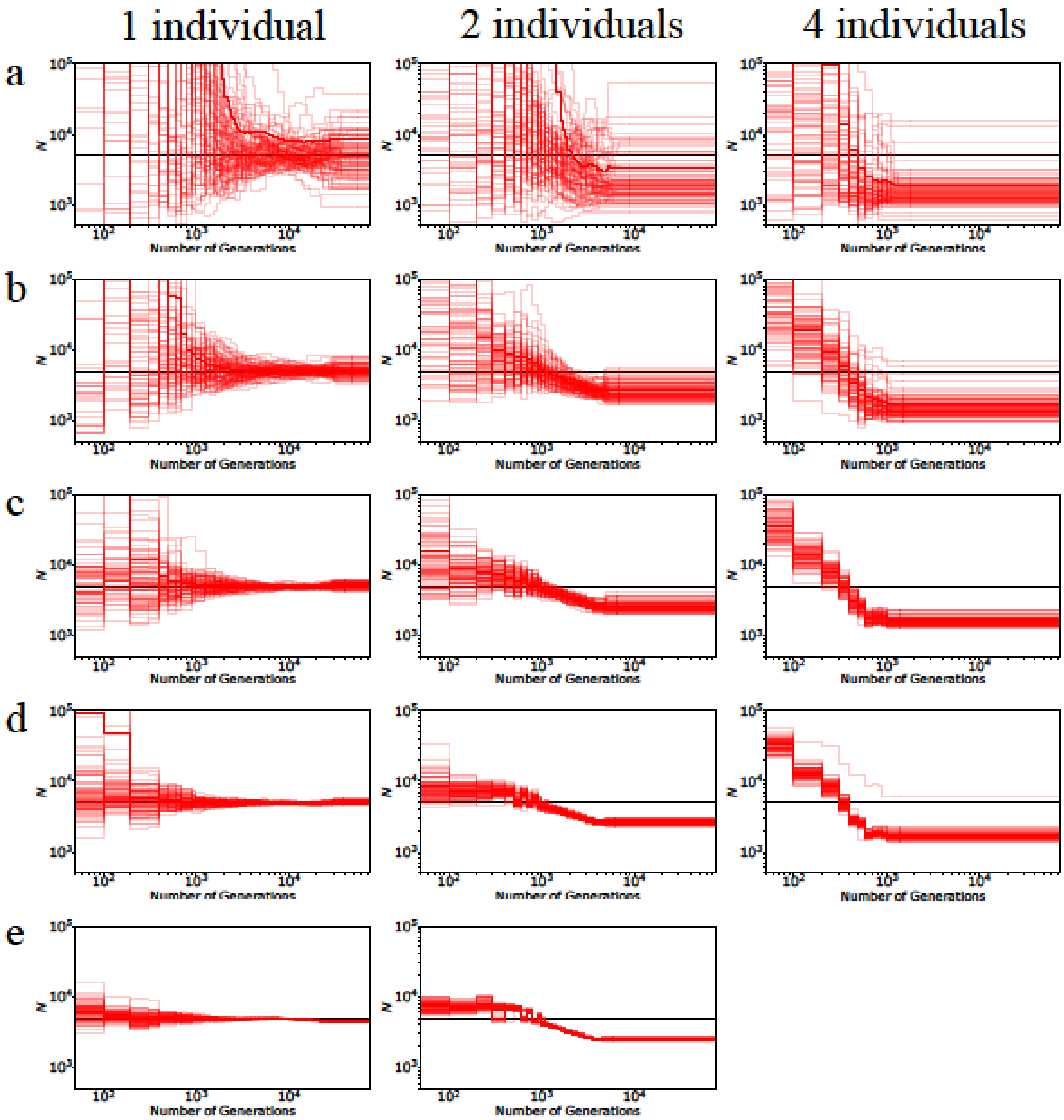

Supp Figure 2: Performance of MSMC under neutrality and demographic equilibrium when using 1, 2, and 4 diploid individuals for inference, for varying chromosome sizes: (a) 1 Mb, (b) 10 Mb, (c) 50 Mb, (d) 200 Mb, (e) 1 Gb. Simulations were performed using msprime (0.7.3) with 100 replicates for each panel. MSMC runs with 4 diploid individuals at 1Gb could not be obtained due to the long computational times required. Detailed methods including command lines can be found here: https://github.com/paruljohri/demographic_inference_with_selection/blob/main/CommandLines/SuppFigure2.txt.

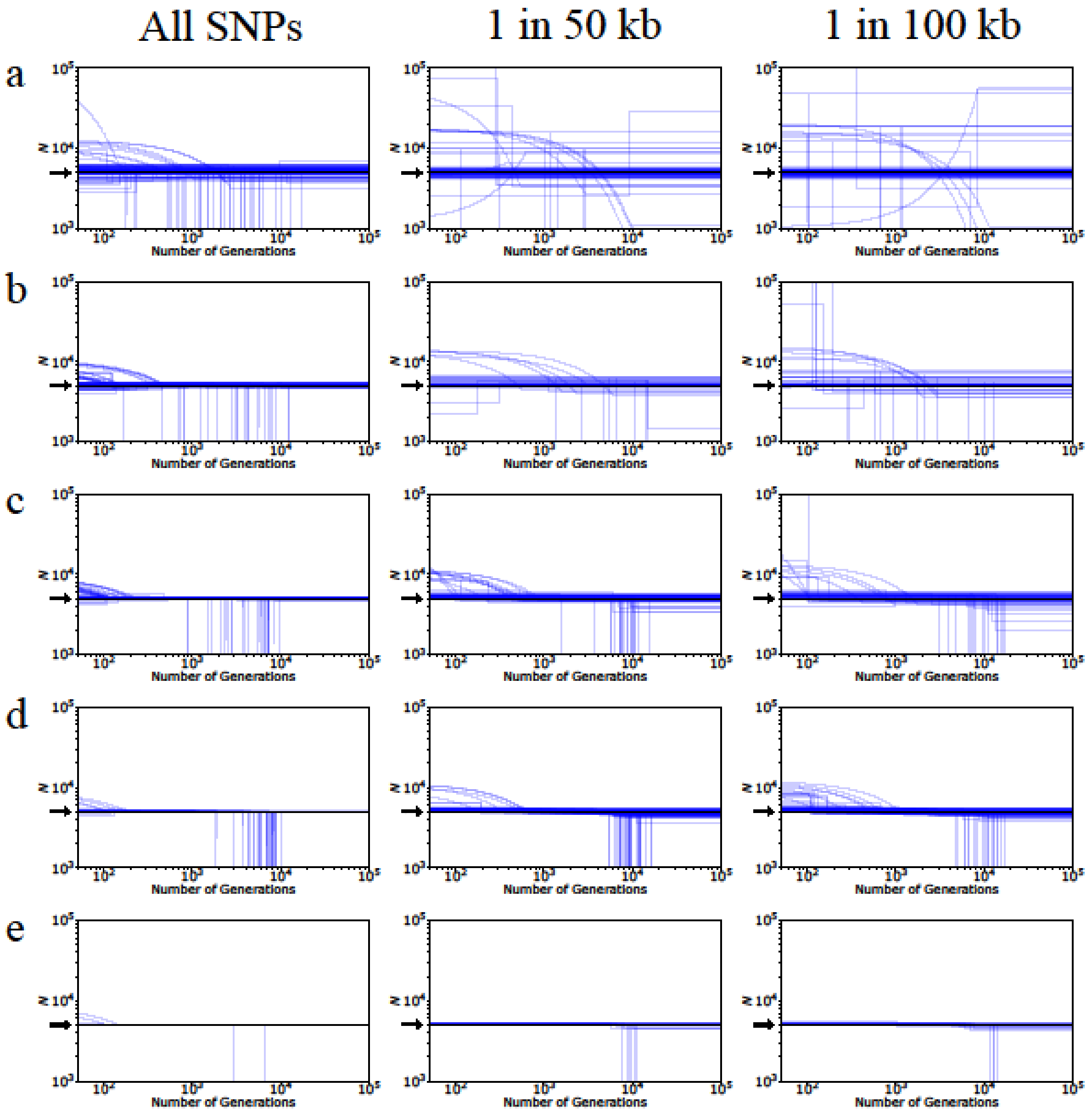

Supp Figure 3: Performance of *fastsimcoal2* under neutrality and demographic equilibrium when simulations were performed using SLiM (3.1) for varying chromosome sizes: (a) 1 Mb, (b) 10 Mb, (c) 50 Mb, (d) 200 Mb, (e) 1 Gb. Model selection was performed with 4 models – equilibrium, instantaneous size change, exponential size change, and instantaneous bottleneck. Inference was performed using 50 diploid individuals using all SNPs (left column), 1 SNP per 50 kb (middle column) and 1 SNP per 100 kb (right column). The inferred population size estimates of the best model are plotted (blue lines). The numbers of replicates for (a)-(d) were 100, while for (e) were 10. The true model is shown in black and indicated by the position of the arrow. Detailed methods including command lines can be found here: https://github.com/paruljohri/demographic_inference_with_selection/blob/main/CommandLines/SuppFigure3_SuppTable2_5.txt.

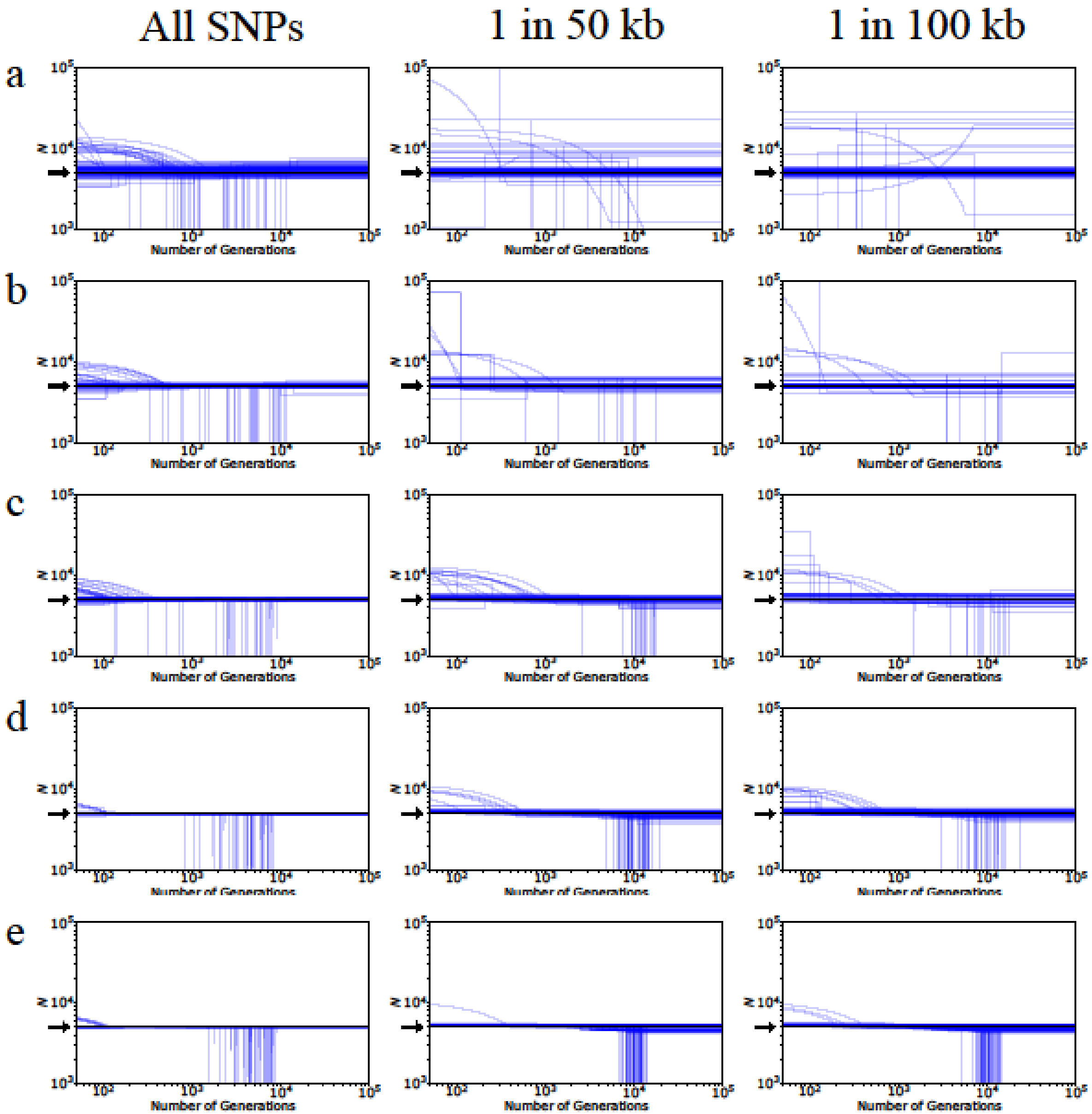

Supp Figure 4: Performance of *fastsimcoal2* under neutrality and demographic equilibrium when simulations were performed using msprime (0.7.3) for varying chromosome sizes: (a) 1 Mb, (b) 10 Mb, (c) 50 Mb, (d) 200 Mb, (e) 1 Gb. Model selection was performed with 4 models – equilibrium, instantaneous size change, exponential size change, and instantaneous bottleneck. Inference was performed using 50 diploid individuals using all SNPs (left column), 1 SNP per 50 kb (middle column) and 1 SNP per 100 kb (right column). The inferred population size estimates of the best model are plotted (blue lines), with 100 replicates for each panel. The true model is shown in black and indicated by the position of the arrow. Detailed methods including command lines can be found here: https://github.com/paruljohri/demographic_inference_with_selection/blob/main/CommandLines/SuppFigure4_SuppTable3_6.txt.

	Functional Density	5%				10%				20%			
True Model	Test Model / DFE Shape	Equilibrium	Exponential Change	Instantaneous change	Instant Bottleneck	Equilibrium	Exponential Change	Instantaneous change	Instant Bottleneck	Equilibrium	Exponential Change	Instantaneous change	Instant Bottleneck
Equilibrium	DFE0	0	1	9	0	0	0	10	0	0	0	10	0
	DFE1	0	1	9	0	0	2	8	0	0	2	8	0
	DFE2	0	0	10	0	0	2	8	0	0	0	10	0
	DFE3	0	6	4	0	0	10	0	0	0	10	0	0
	DFE4	0	1	9	0	0	0	10	0	0	9	1	0
	DFE5	0	6	4	0	0	10	0	0	0	10	0	0
	DFE6	0	6	4	0	0	6	4	0	0	10	0	0
Instantaneous Decline (6x)	DFE0	0	0	10	0	0	0	10	0	0	0	10	0
	DFE1	0	0	10	0	0	0	10	0	0	0	10	0
	DFE2	0	0	10	0	0	0	10	0	0	0	0	10
	DFE3	0	0	10	0	0	10	0	0	0	10	0	0
	DFE4	0	0	10	0	0	0	10	0	0	10	0	0
	DFE5	0	0	10	0	0	0	0	10	0	10	0	0
	DFE6	0	0	10	0	0	0	10	0	0	10	0	0
Instantaneous Decline (2x)	DFE0	0	0	10	0	0	0	10	0	0	0	10	0
	DFE1	0	0	10	0	0	0	10	0	0	0	10	0
	DFE2	0	0	10	0	0	0	10	0	0	0	0	10
	DFE3	0	0	10	0	0	0	0	10	0	10	0	0
	DFE4	0	0	10	0	0	0	10	0	0	0	0	10
	DFE5	0	0	10	0	0	0	10	0	0	10	0	0
	DFE6	0	0	10	0	0	0	10	0	0	0	0	10
Exponential Growth (30x)	DFE0	0	10	0	0	0	10	0	0	0	10	0	0
	DFE1	0	10	0	0	0	10	0	0	0	10	0	0
	DFE2	0	10	0	0	0	10	0	0	0	10	0	0
	DFE3	0	10	0	0	0	10	0	0	0	10	0	0
	DFE4	0	10	0	0	0	10	0	0	0	10	0	0
	DFE5	0	10	0	0	0	10	0	0	0	10	0	0
	DFE6	0	10	0	0	0	10	0	0	0	10	0	0
Exponential Growth (2x)	DFE0	0	0	10	0	0	0	10	0	0	0	10	0
	DFE1	0	0	10	0	0	0	10	0	0	0	10	0
	DFE2	0	0	10	0	0	0	10	0	0	0	10	0
	DFE3	0	0	10	0	0	0	10	0	0	0	10	0
	DFE4	0	0	10	0	0	0	10	0	0	0	10	0
	DFE5	0	0	10	0	0	0	10	0	0	0	10	0
	DFE6	0	0	10	0	0	0	10	0	0	0	10	0

Supp Figure 5: Model selection by *fastsimcoal2* in the presence of BGS, when chosen from four possible models: equilibrium, instantaneous size change, exponential size change, and instantaneous bottleneck. The DFEs are specified in Table 1, and DFE0 refers to neutrality. Results are shown when all SNPs were used for inference, when 5%, 10% or 20% of the genomes were exonic (with exonic sites masked), and the standard AIC penalty was used. Detailed methods including command lines can be found here: https://github.com/paruljohri/demographic_inference_with_selection/blob/main/CommandLines/SuppFigure5.txt.

AIC penalty	True Model	Test Model / DFE Shape	Equilibrium	Exponential Change	Instantaneous change	Instant Bottleneck
2x	Equilibrium	DFE0	0	0	8	2
		DFE1	0	0	10	0
		DFE2	0	1	9	0
		DFE3	0	10	0	0
		DFE4	0	3	7	0
		DFE5	0	10	0	0
		DFE6	0	6	4	0
	Instantaneous Decline (6x)	DFE0	0	0	10	0
		DFE1	0	0	0	10
		DFE2	0	10	0	0
		DFE3	0	10	0	0
		DFE4	0	10	0	0
		DFE5	0	10	0	0
		DFE6	0	10	0	0
	Exponential Growth (30x)	DFE0	0	10	0	0
		DFE1	0	10	0	0
		DFE2	0	10	0	0
		DFE3	0	10	0	0
		DFE4	0	10	0	0
		DFE5	0	10	0	0
		DFE6	0	10	0	0
25x	Equilibrium	DFE0	10	0	0	0
		DFE1	0	0	10	0
		DFE2	0	1	9	0
		DFE3	0	10	0	0
		DFE4	0	3	7	0
		DFE5	0	10	0	0
		DFE6	0	6	4	0
	Instantaneous Decline (6x)	DFE0	0	0	10	0
		DFE1	0	0	0	10
		DFE2	0	10	0	0
		DFE3	0	10	0	0
		DFE4	0	10	0	0
		DFE5	0	10	0	0
		DFE6	0	10	0	0
	Exponential Growth (30x)	DFE0	0	10	0	0
		DFE1	0	10	0	0
		DFE2	0	10	0	0
		DFE3	0	10	0	0
		DFE4	0	10	0	0
		DFE5	0	10	0	0
		DFE6	0	10	0	0

Supp Figure 6: Model selection by *fastsimcoal2* in the presence of BGS, when chosen from four possible models: equilibrium, instantaneous size change, exponential size change, and instantaneous bottleneck. The DFEs are specified in Table 1, and DFE0 refers to neutrality. Results are shown when SNPs used for inference were separated at a distance of 100 kb, 20% of the genomes were exonic, and the AIC penalty was 2× (standard) or 25× the number of parameters. Detailed methods including command lines can be found here: https://github.com/paruljohri/demographic_inference_with_selection/blob/main/CommandLines/SuppFigure6.txt.

	Functional Density	5%			10%			20%		
True Model	Behavior / DFE Shape	Growth	Decline	Bottleneck	Growth	Decline	Bottleneck	Growth	Decline	Bottleneck
Equilibrium	DFE1	10	0	0	10	0	0	10	0	0
	DFE2	10	0	0	10	0	0	10	0	0
	DFE3	10	0	0	10	0	0	10	0	0
	DFE4	10	0	0	10	0	0	10	0	0
	DFE5	10	0	0	10	0	0	10	0	0
	DFE6	10	0	0	10	0	0	10	0	0
Instantaneous Decline (6x)	DFE1	0	10	0	0	10	0	0	10	0
	DFE2	0	10	0	0	10	0	0	0	10
	DFE3	0	10	0	10	0	0	10	0	0
	DFE4	0	10	0	0	10	0	10	0	0
	DFE5	0	10	0	0	0	10	10	0	0
	DFE6	0	10	0	0	10	0	10	0	0
Instantaneous Decline (2x)	DFE1	0	10	0	0	10	0	0	10	0
	DFE2	0	10	0	0	10	0	0	0	10
	DFE3	0	10	0	0	0	10	10	0	0
	DFE4	0	10	0	0	10	0	0	0	10
	DFE5	0	10	0	0	10	0	10	0	0
	DFE6	0	10	0	0	10	0	0	0	10
Exponential Growth (30x)	DFE1	10	0	0	10	0	0	10	0	0
	DFE2	10	0	0	10	0	0	10	0	0
	DFE3	10	0	0	10	0	0	10	0	0
	DFE4	10	0	0	10	0	0	10	0	0
	DFE5	10	0	0	10	0	0	10	0	0
	DFE6	10	0	0	10	0	0	10	0	0
Exponential Growth (2x)	DFE1	10	0	0	10	0	0	10	0	0
	DFE2	10	0	0	10	0	0	10	0	0
	DFE3	10	0	0	10	0	0	10	0	0
	DFE4	10	0	0	10	0	0	10	0	0
	DFE5	10	0	0	10	0	0	10	0	0
	DFE6	10	0	0	10	0	0	10	0	0

Supp Figure 7: Effects of BGS on inference of growth or decline by *fastsimcoal2*. The inferred model was classified as growth if $N_{anc} < N_{cur}$ and as decline if $N_{anc} > N_{cur}$. The DFEs are specified in Table 1. Results are shown for all SNPs, when 5%, 10% and 20% of the genomes were exonic (with exonic sites masked), and a standard AIC penalty was used. Detailed methods including command lines can be found here: https://github.com/paruljohri/demographic_inference_with_selection/blob/main/CommandLines/SuppFigure7.txt.

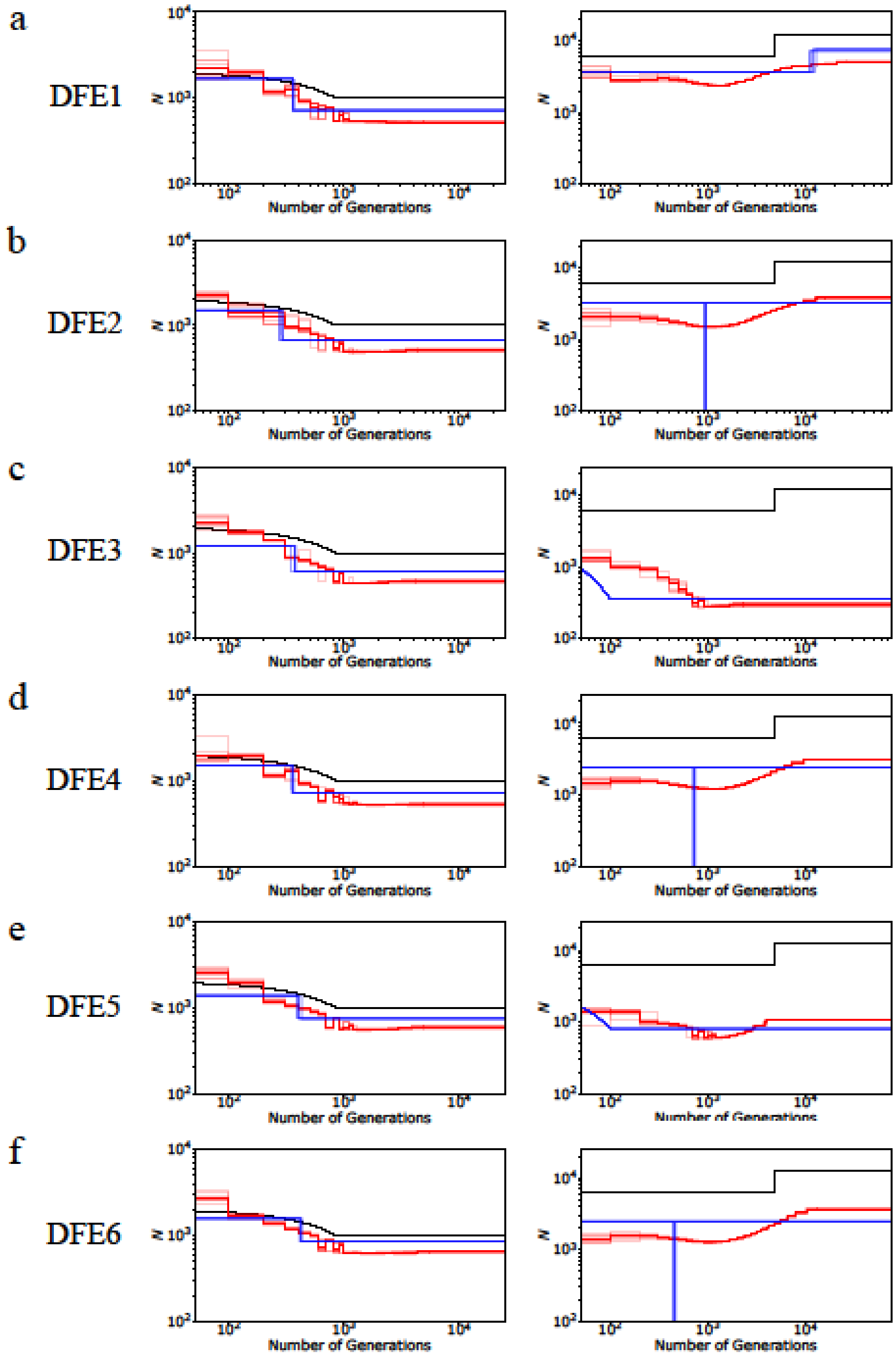

Supp Figure 8: Inferred demography from MSMC (red lines) and *fastsimcoal2* (blue lines) in the presence of background selection, with the true DFE shown to the left of the panel, for 2-fold instantaneous decline (right column) and 2-fold exponential growth (left column). In this case, 20% of the genome was exonic (and exonic sites were masked). The true demographic models are shown as black lines. Detailed methods including command lines can be found here: https://github.com/paruljohri/demographic_inference_with_selection/blob/main/CommandLines/SuppFigure8.txt.

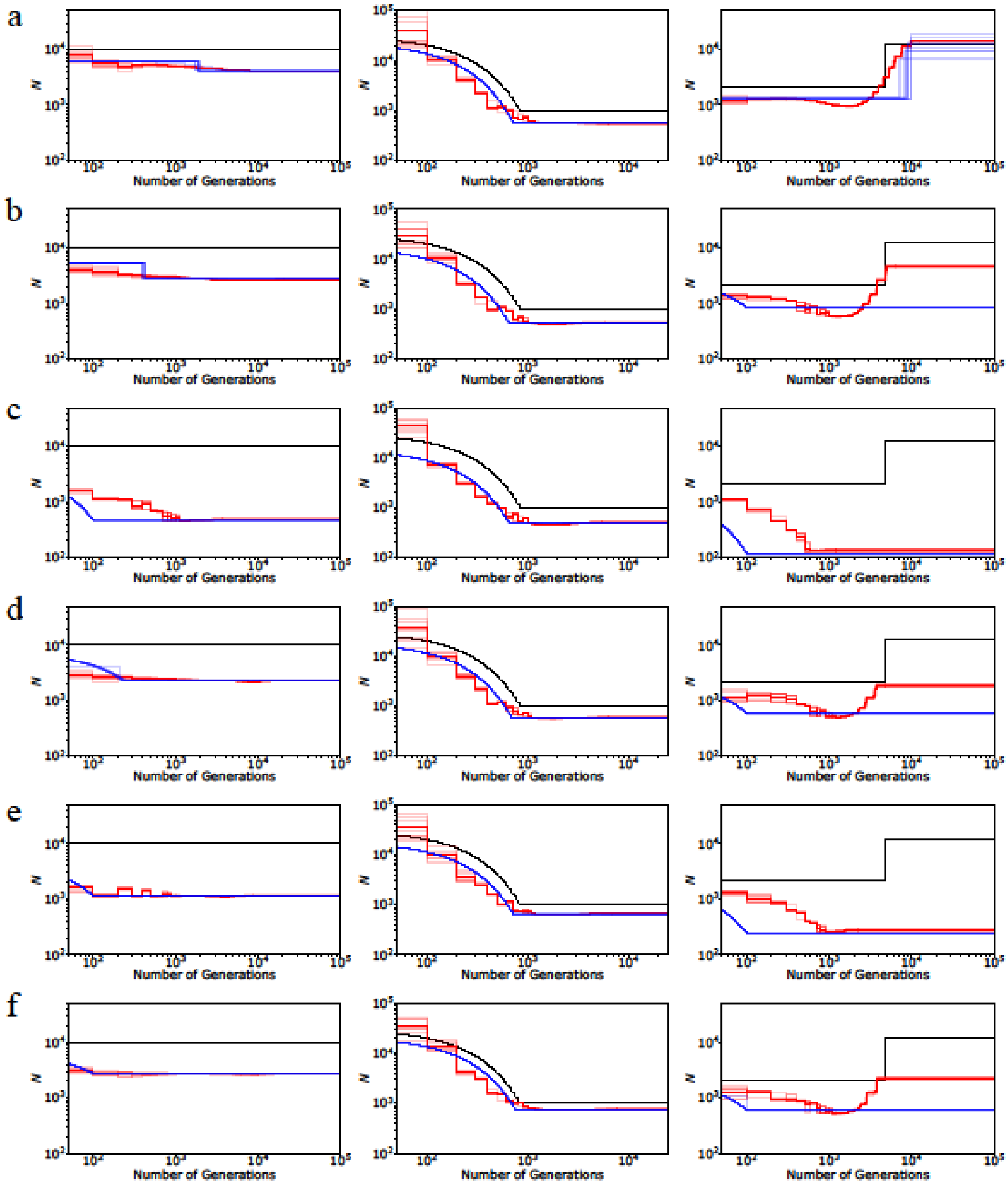

Supp Figure 9: Inference of demography by MSMC (red lines; 10 replicates) and *fastsimcoal2* (blue lines; 10 replicates) under demographic equilibrium (left column), 30-fold exponential growth (middle column), and ~6-fold instantaneous decline (right column) in the presence of direct purifying selection (*i.e.*, directly selected sites are not masked). The true demographic model is depicted in black lines. Exonic sites experience purifying selection specified by the following DFEs (defined in Table 1): (a) DFE1, (b) DFE2, (c) DFE3, (d) DFE4, (e) DFE5, (f) DFE6. In this case, 20% of the genome was exonic and all SNPs were used for inference, including exonic sites. Detailed methods including command lines can be found here: https://github.com/paruljohri/demographic_inference_with_selection/blob/main/CommandLines/SuppFigure9.txt.

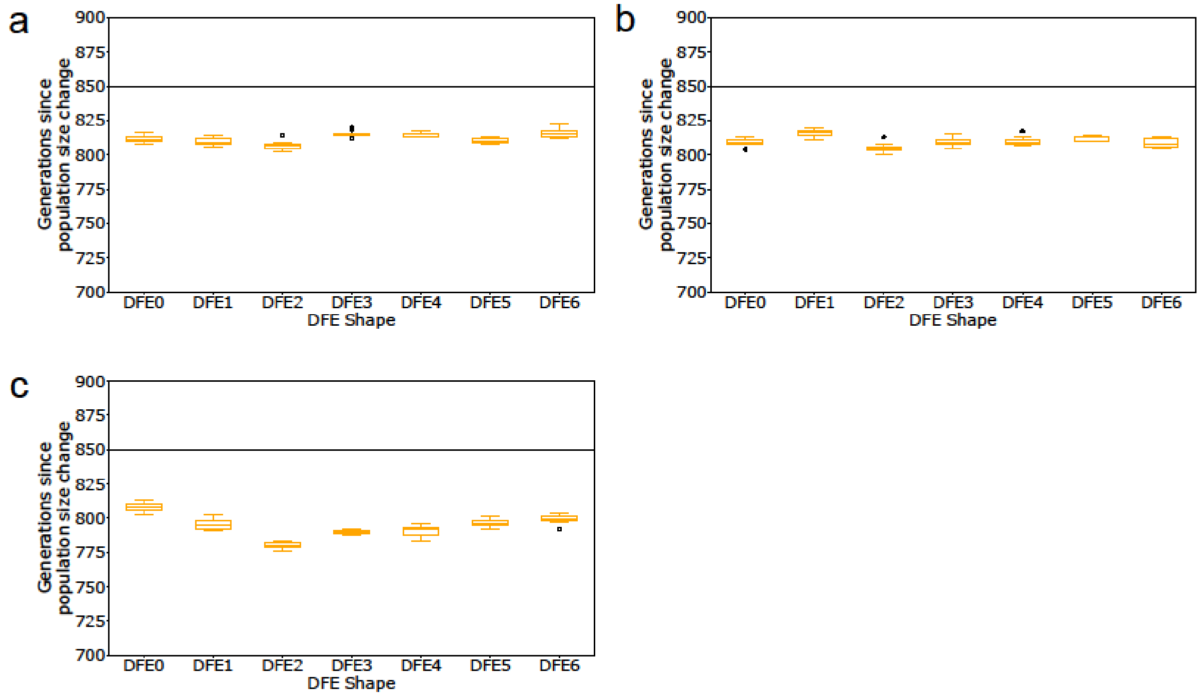

Supp Figure 10: Inference of the timing of 30-fold exponential growth by *fastsimcoal2* in the presence of BGS compared to neutrality (DFE0). The DFEs are specified in Table 1. Results are shown for the case where (a) 5%, (b) 10%, and (c) 20% of the genome is exonic (with exonic sites masked), and all non-exonic SNPs are used for inference. The black horizontal line represents the true timing.

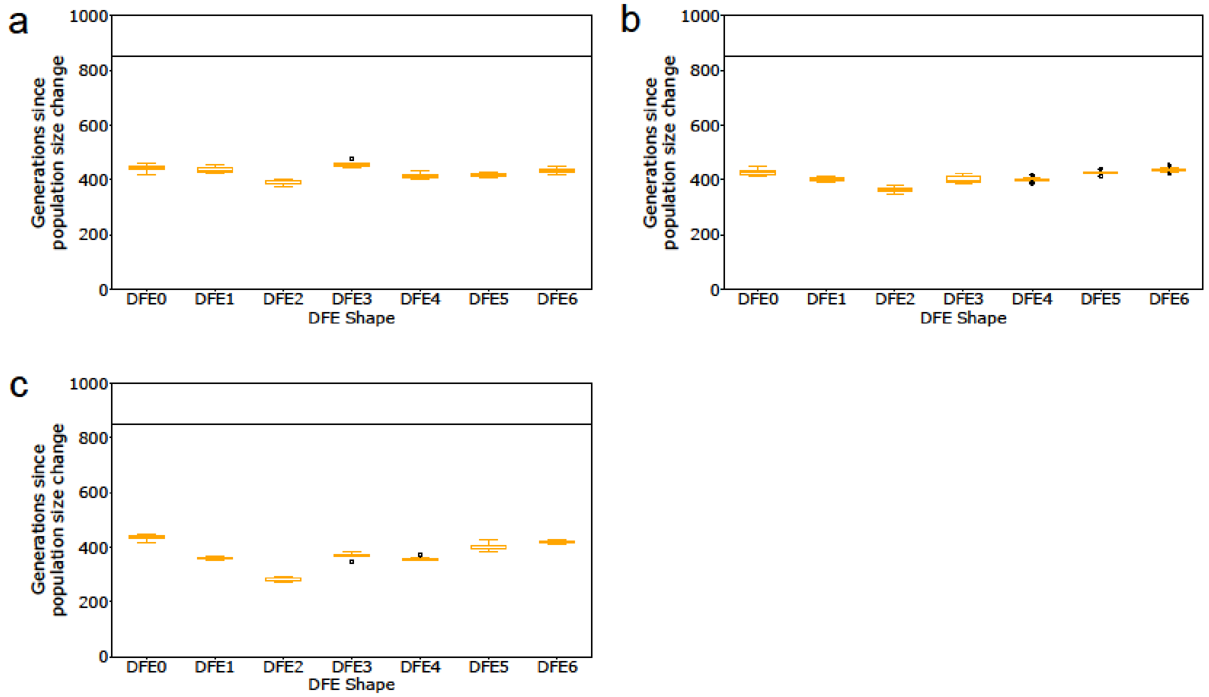

Supp Figure 11: Inference of the timing of 2-fold exponential growth by *fastsimcoal2* in the presence of BGS compared to neutrality (DFE0). Results are shown for the case where (a) 5%, (b) 10%, and (c) 20% of the genome is exonic (with exonic sites masked), and all non-exonic SNPs are used for inference. The black horizontal line represents the true timing.

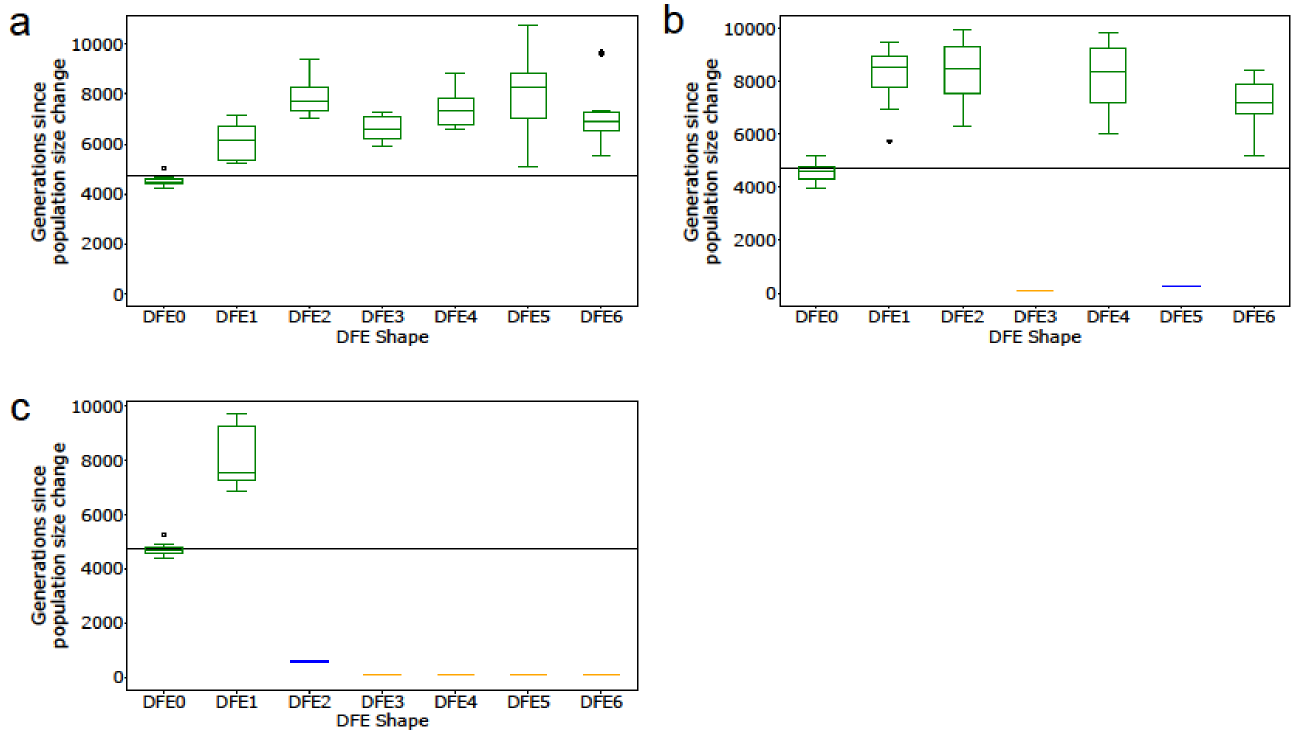

Supp Figure 12: Inference of the timing of 6-fold instantaneous decline by *fastsimcoal2* in the presence of BGS compared to neutrality (DFE0). The DFEs are specified in Table 1. Results are shown for the case in which (a) 5%, (b) 10%, and (c) 20% of the genome is exonic (with exonic sites masked), and all non-exonic SNPs are used for inference. The black horizontal line represents the true timing. Boxplots are presented in green if decline was inferred, in yellow if growth was inferred, and in blue if a bottleneck was inferred.

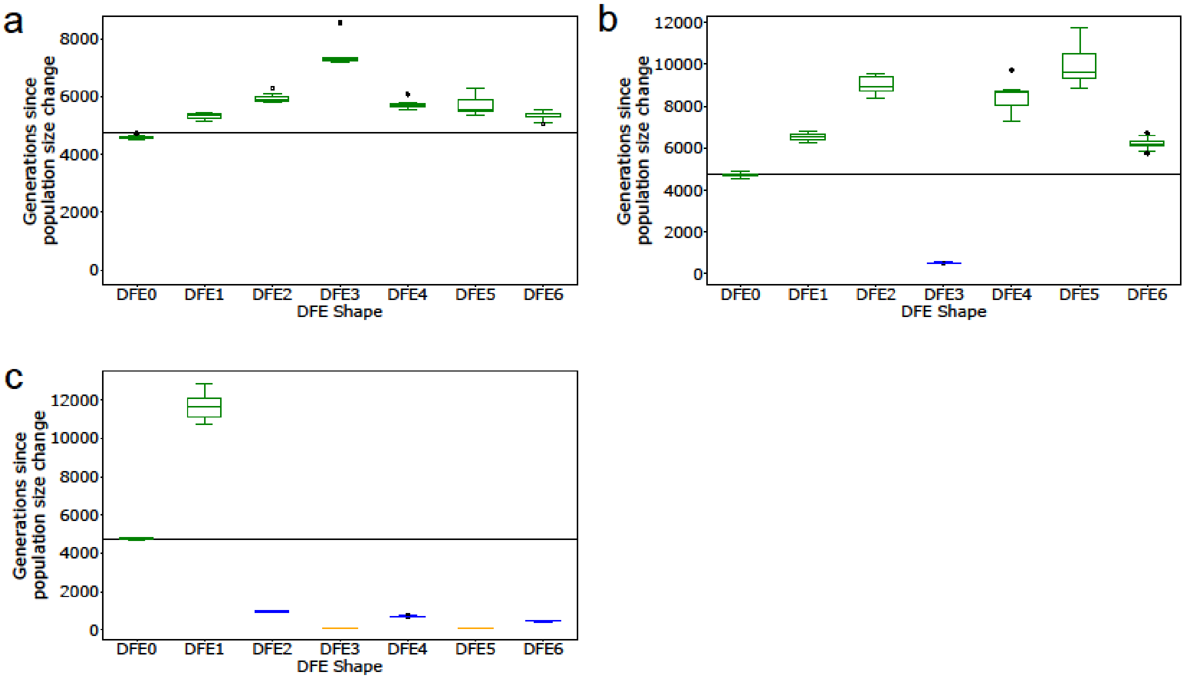

Supp Figure 13: Inference of the timing of 2-fold instantaneous decline by *fastsimcoal2* in the presence of BGS compared to neutrality (DFE0). The DFEs are specified in Table 1. Results are shown for the case where (a) 5%, (b) 10%, and (c) 20% of the genome is exonic (with exonic sites masked), and all non-exonic SNPs are used for inference. The black horizontal line represents the true timing. Boxplots are presented in green if decline was inferred, in yellow if growth was inferred, and in blue if a bottleneck was inferred.

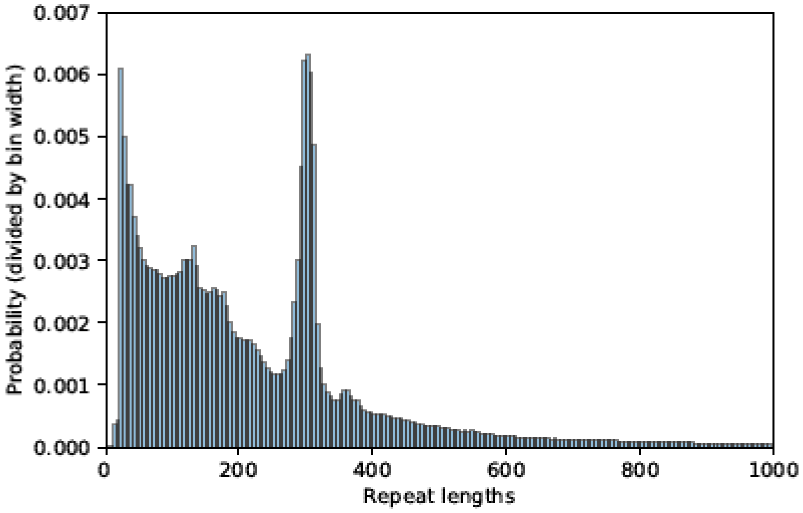

Supp Figure 14: Distribution of lengths of repeat regions in the human genomes (*hg19*). Shown above is the distribution of lengths up to 1000 bp, although lengths of repeat regions range between 6 to 160602 bp. Detailed methods including command lines can be found here: https://github.com/paruljohri/demographic_inference_with_selection/blob/main/CommandLines/SuppFigure14.txt.

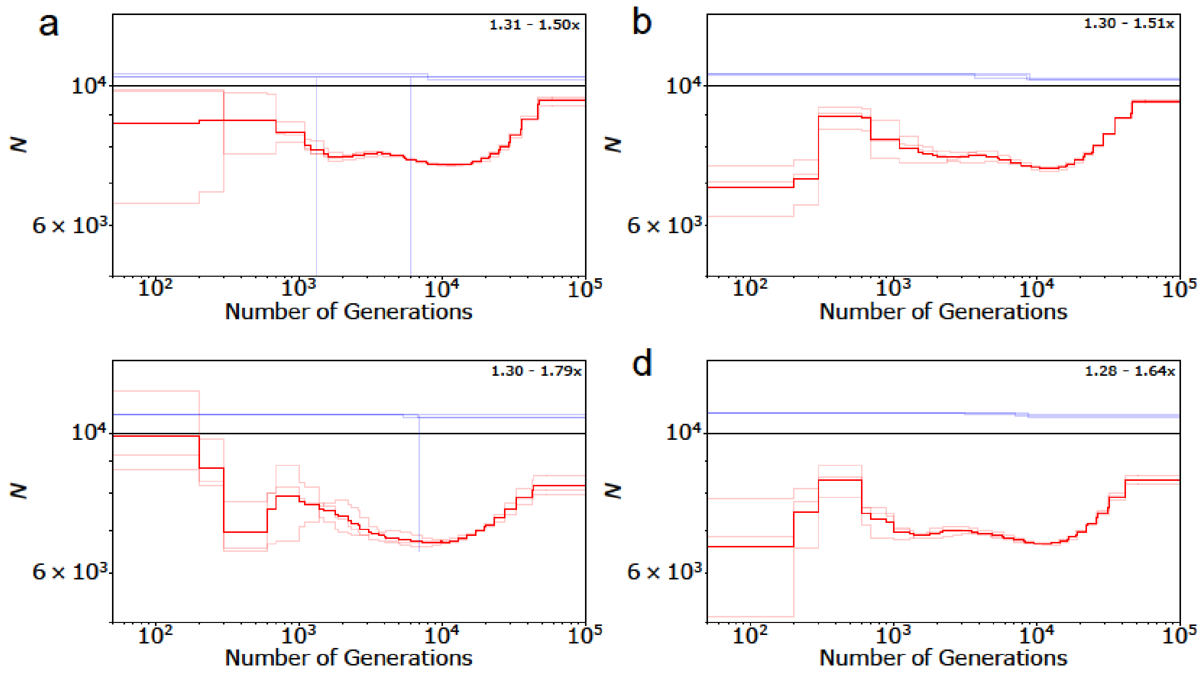

Supp Figure 15: Performance of demographic inference by MSMC (red lines) and *fastsimcoal2* (blue lines) under different scenarios of neutrality, when the true model is equilibrium: (a) there is variation in recombination and mutation rates, (b) there is variation in recombination and mutation rates and the centromeric region is masked, (c) there is variation in recombination and mutation rates, and short regions resembling repeats (comprising 10% of each chromosome) are randomly masked across the genome, and (d) there is variation in recombination and mutation rates, and the centromere as well as small-sized repeats are randomly masked across the genome. The maximum and minimum fold change detected in every scenario is indicated on the upper right corner. Detailed methods including command lines can be found here: https://github.com/paruljohri/demographic_inference_with_selection/blob/main/CommandLines/SuppFigure15-20.txt.

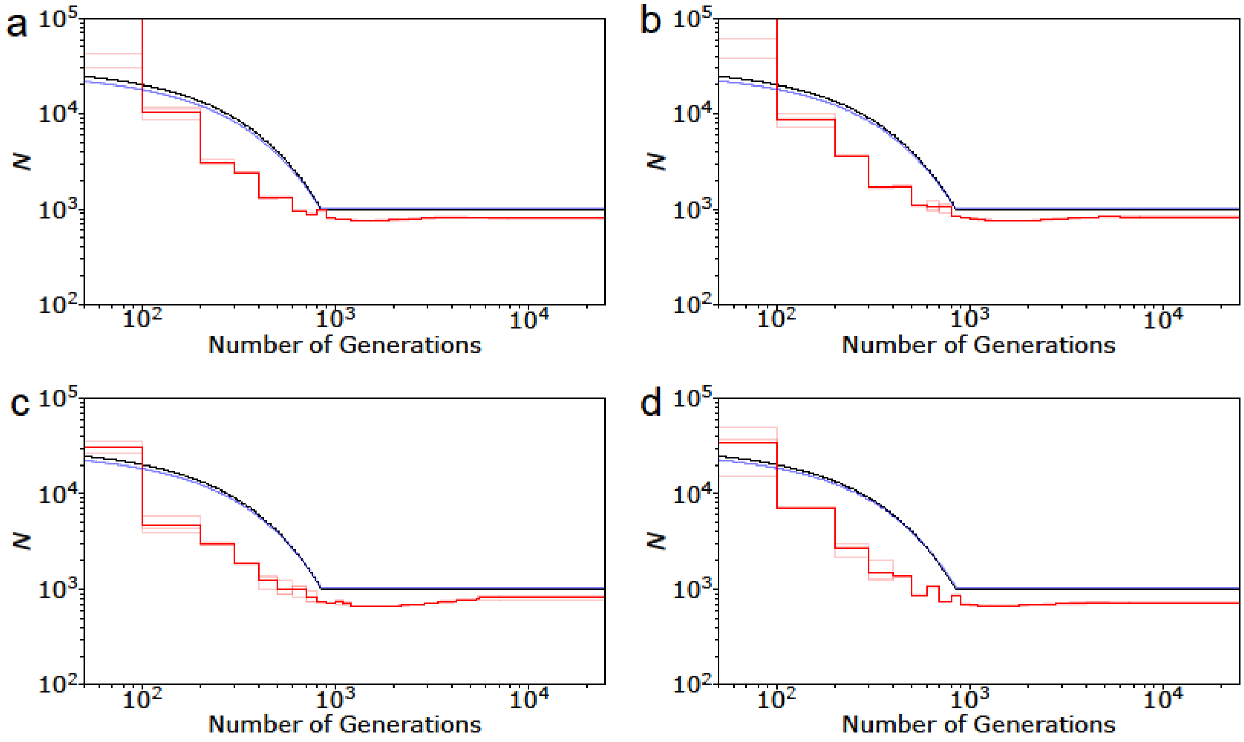

Supp Figure 16 : Performance of demographic inference by MSMC (red lines) and *fastsimcoal2* (blue lines) under different scenarios of neutrality, when the true model is 30-fold exponential growth: (a) there is variation in recombination and mutation rates across the genome, (b) there is variation in recombination and mutation rates, and the centromeric region is masked, (c) there is variation in recombination and mutation rates, and short regions resembling repeats are randomly masked across the genome (comprising of 10% of each chromosome), and (d) there is variation in recombination and mutation rates, and the centromere as well as small-sized repeats are randomly masked across the genome. Detailed methods including command can be found here: https://github.com/paruljohri/demographic_inference_with_selection/blob/main/CommandLines/SuppFigure15-20.txt.

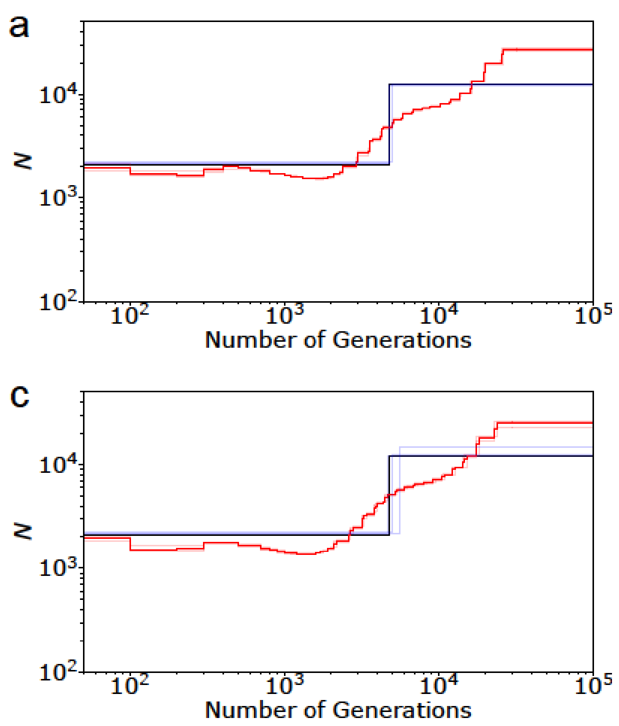

Supp Figure 17: Performance of demographic inference by MSMC (red lines) and *fastsimcoal2* (blue lines) under different scenarios of neutrality, when the true model is 6-fold instantaneous decline: (a) there is variation in recombination and mutation rates across the genome, (b) there is variation in recombination and mutation rates, and the centromeric region is masked, (c) there is variation in recombination sand mutation rates, and short regions resembling repeats are randomly masked across the genome (comprising of 10% of each chromosome), and (d) there is variation in recombination and mutation rates, and the centromere as well as small-sized repeats are randomly masked across the genome. Detailed methods including command lines can be found here:
https://github.com/paruljohri/demographic_inference_with_selection/blob/main/CommandLines/SuppFigure15-20.txt.

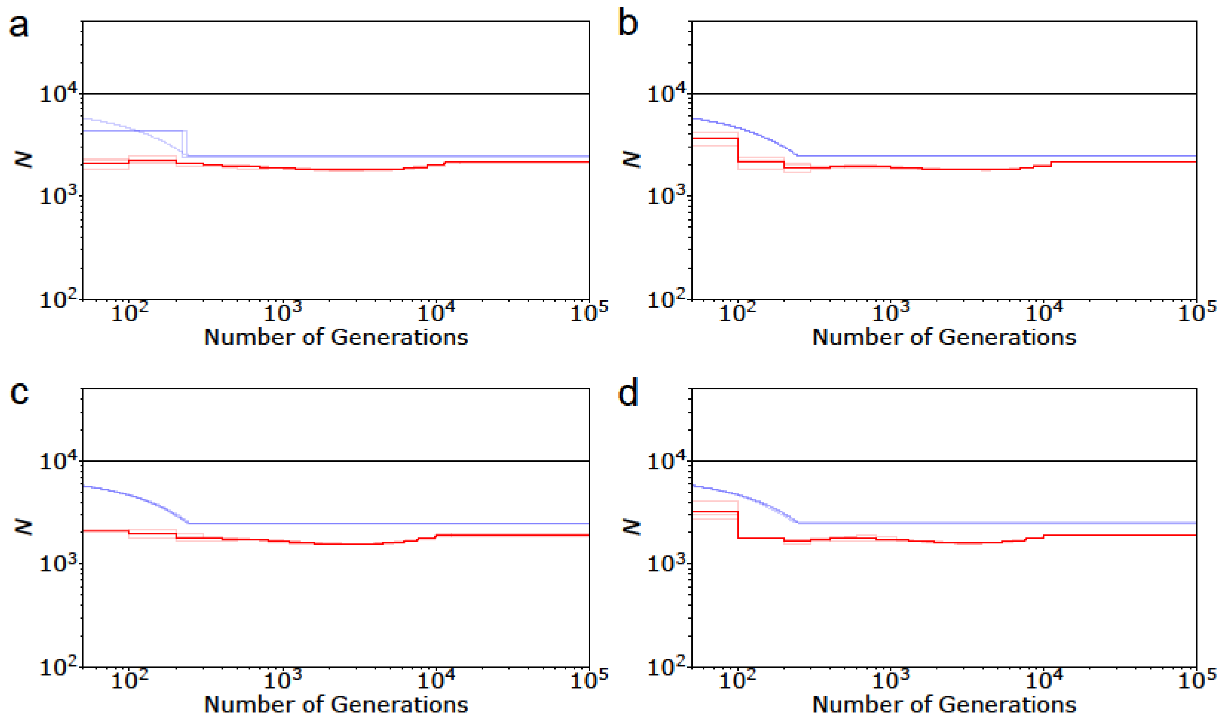

Supp Figure 18: Performance of demographic inference by MSMC (red lines) and *fastsimcoal2* (blue lines) in the presence of background selection, under different scenarios when the true model is equilibrium: (a) there is variation in recombination and mutation rates, (b) there is variation in recombination and mutation rates and the centromeric region is masked, (c) there is variation in recombination and mutation rates, and short regions resembling repeats (comprising 10% of each chromosome) are randomly masked across the genome, and (d) there is variation in recombination and mutation rates, and the centromere as well as small-sized repeats are randomly masked across the genome. Exons comprise of 20% of the genome, experience purifying selection given by DFE4 ($f_0 = f_1 = f_2 = f_3 = 0.25$), and are masked when performing inference. Detailed methods including command lines can be found here: https://github.com/paruljohri/demographic_inference_with_selection/blob/main/CommandLines/SuppFigure15-20.txt.

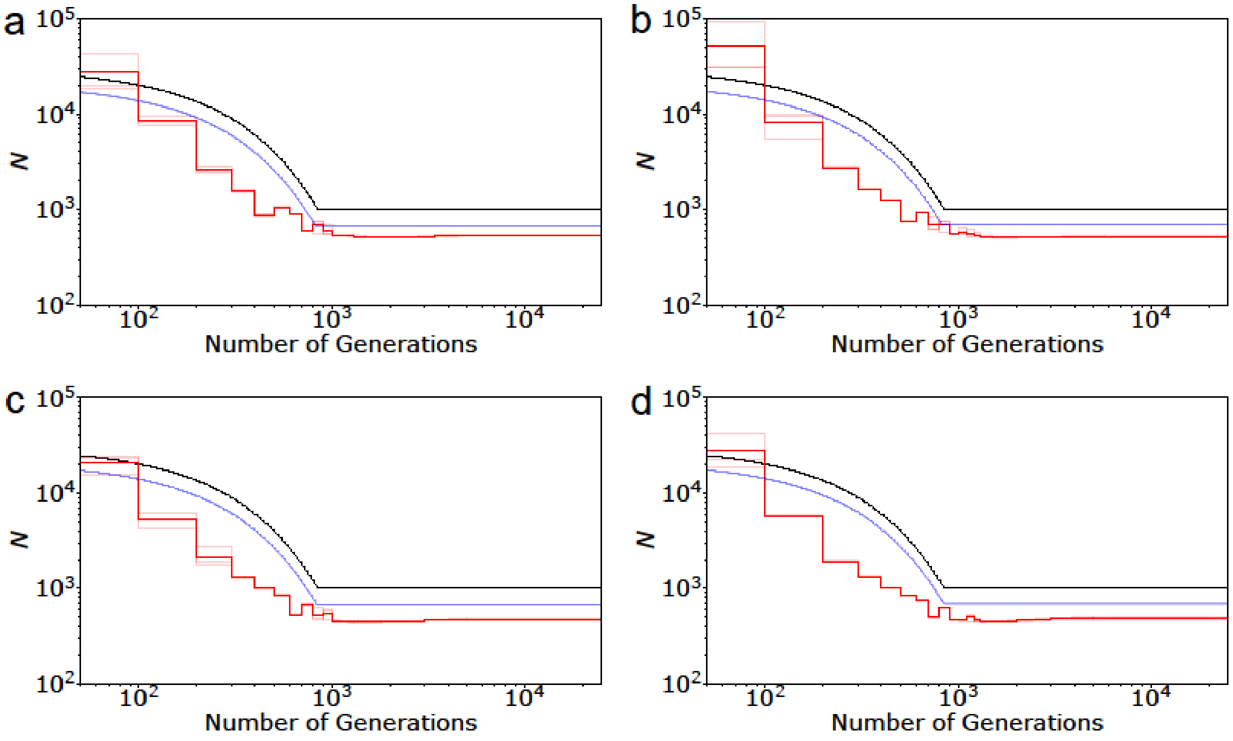

Supp Figure 19: Performance of demographic inference by MSMC (red lines) and *fastsimcoal2* (blue lines) in the presence of background selection, under different scenarios when the true model is 30-fold exponential growth: (a) there is variation in recombination and mutation rates, (b) there is variation in recombination and mutation rates and the centromeric region is masked, (c) there is variation in recombination and mutation rates, and short regions resembling repeats (comprising 10% of each chromosome) are randomly masked across the genome, and (d) there is variation in recombination and mutation rates, and the centromere as well as small-sized repeats are randomly masked across the genome. Exons comprise of 20% of the genome, experience purifying selection given by DFE4 ($f_0 = f_1 = f_2 = f_3 = 0.25$), and are masked when performing inference. Detailed methods including command lines can be found here: https://github.com/paruljohri/demographic_inference_with_selection/blob/main/CommandLines/SuppFigure15-20.txt.

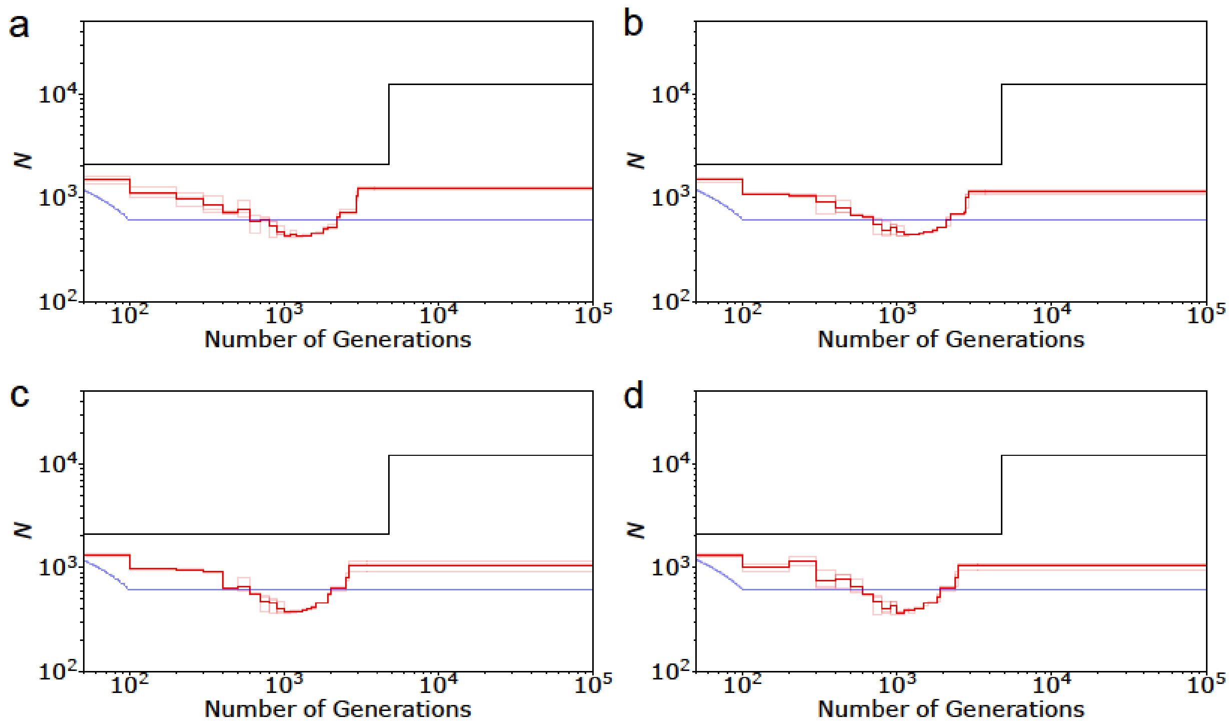

Supp Figure 20: Performance of demographic inference by MSMC (red lines) and *fastsimcoal2* (blue lines) in the presence of background selection, under different scenarios when the true model is a 6-fold instantaneous decline: (a) there is variation in recombination and mutation rates, (b) there is variation in recombination and mutation rates and the centromeric region is masked, (c) there is variation in recombination and mutation rates, and short regions resembling repeats (comprising 10% of each chromosome) are randomly masked across the genome, and (d) there is variation in recombination and mutation rates, and the centromere as well as small-sized repeats are randomly masked across the genome. Exons comprise of 20% of the genome, experience purifying selection given by DFE4 ($f_0 = f_1 = f_2 = f_3 = 0.25$), and are masked when performing inference. Detailed methods including command lines can be found here: https://github.com/paruljohri/demographic_inference_with_selection/blob/main/CommandLines/SuppFigure15-20.txt.

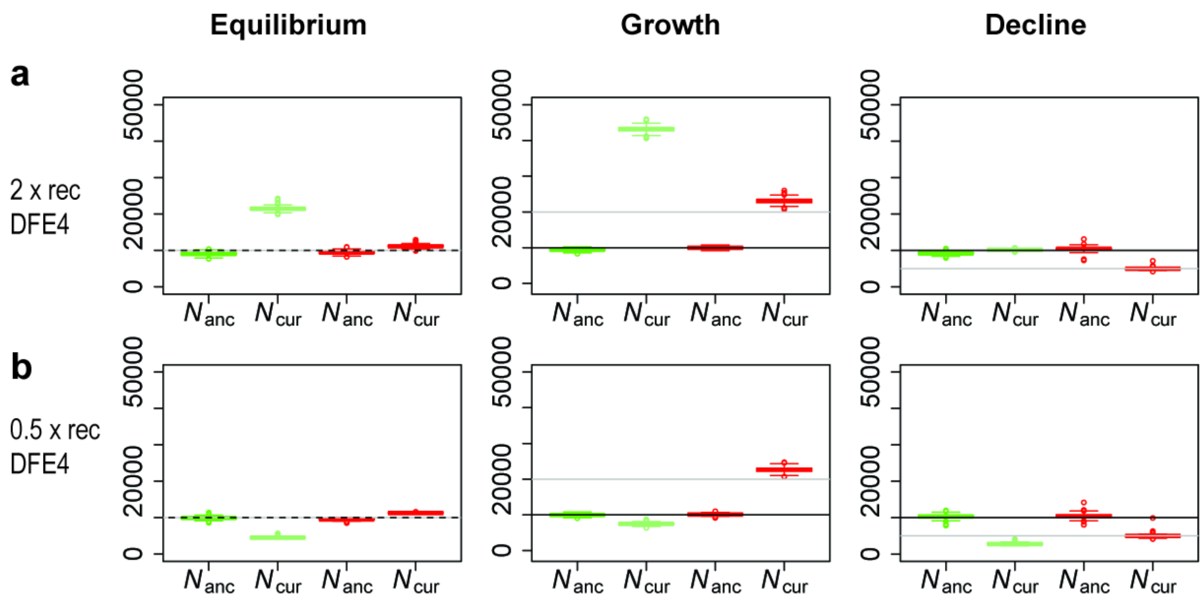

Supp Figure 21: Performance of the ABC method when recombination rate is mis-specified. (a) The true recombination rate is 2-fold higher than that assumed, and (b) the true recombination rate is 2-fold lower than that assumed. Boxplots in green show the posterior estimates when the recombination rate is higher or lower than assumed. For comparison, boxplots in red show the posterior inferred when the corresponding recombination rate is correctly specified. The black line displays the true ancestral population size (N_{anc}) and the gray line represents the true current population size (N_{cur}). Detailed methods including command lines can be found here: https://github.com/paruljohri/demographic_inference_with_selection/blob/main/CommandLines/SuppFigure21.txt.

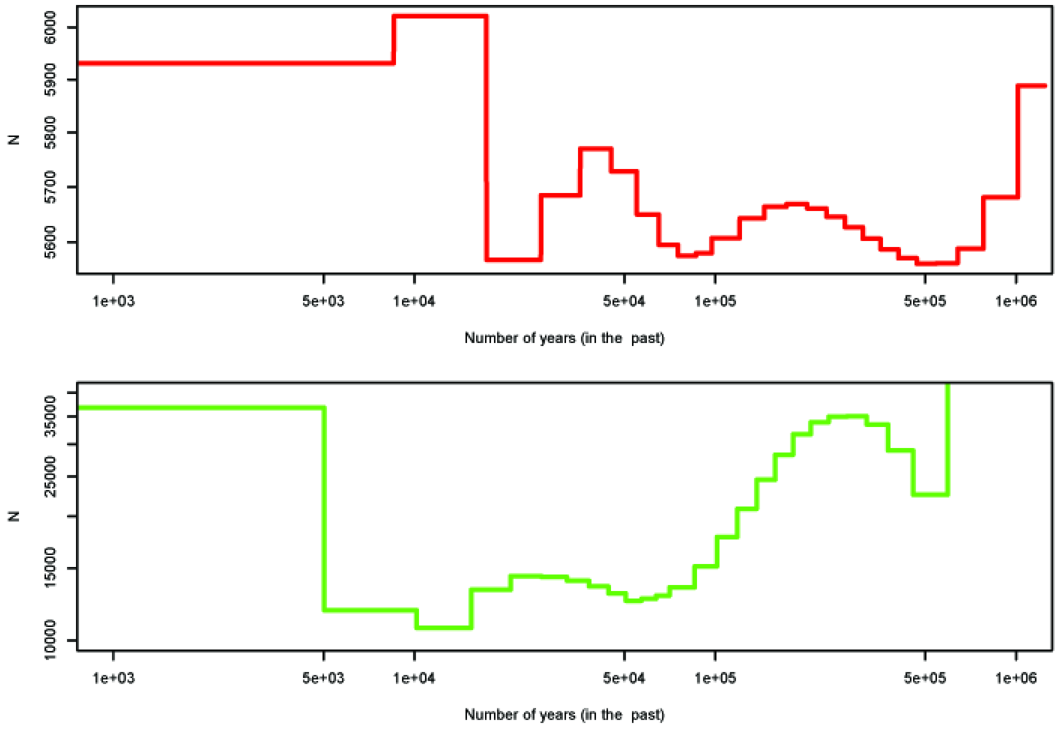

Supp Figure 22: Inference of demographic history by MSMC. Top panel / red line: simulations in which the true model is constant population size, and 50% of new mutations in exons are strongly deleterious with the remainder being neutral, where exons comprise 5% of the genome. Bottom panel / green line: the empirical estimate of population history of the YRI population inferred with MSMC by Schiffels and Durbin (2014). The x-axis is in years (assuming a generation time of 30 years). Note that the y-axes are on different scales, and the magnitude of change observed in the empirical data is considerably larger in the simulated data. Thus, this comparison is only meant to illustrate this common shape taken in MSMC plots (and see similar shapes in, for example, vervets (Warren *et al.* 2015; Figure 4) and passenger pigeons (Hung *et al.* 2014; Figure 2)).
